# Brainstem Hemorrhage After Extradural Hematoma Evacuation: Myth or Truth?

**DOI:** 10.7759/cureus.78375

**Published:** 2025-02-02

**Authors:** Dattatraya Mallik, Jeevesh Mallik, Niraj K Choudhary, Manoj Kumar

**Affiliations:** 1 Neurosurgery, Tata Main Hospital, Jamshedpur, IND

**Keywords:** craniotomy, duret hemorrhage, epidural hematoma, extradural hematoma, postoperative complications

## Abstract

Brainstem hemorrhage can occur as a primary or secondary event in traumatic brain injury (TBI). Duret hemorrhage is a type of brainstem hemorrhage caused by a secondary increase in intracranial pressure and transtentorial herniation. Duret hemorrhage following TBI has been considered an irreversible and terminal event. We report a young adult patient with bifrontal extradural hematoma who presented with a low Glasgow Coma Scale score and advanced signs of cerebral herniation. The patient underwent an urgent craniotomy for evacuation of an acute epidural hematoma and remained in a poor neurological state post-surgery. A postoperative scan was done within 24 hours, which showed a newly developed Duret hemorrhage. The patient was nursed postoperatively with aggressive neurocritical and neurorehabilitation maneuvers; however, continued to have a poor neurological score. Although Duret hemorrhage has been reported in neurosurgical literature owing to various other etiologies, interestingly only four cases till now have been reported post craniotomy for extradural hematoma evacuation. Duret hemorrhage in the setting of cerebral herniation after a severe TBI has been considered a terminal brainstem event with a high incidence of death or persistent vegetative outcome.

## Introduction

Duret hemorrhages, named after the French neuroscientist Henry Duret, are secondary brainstem hemorrhages due to descending transtentorial herniation. Such herniation may occur due to shifts in the intracranial compartments owing to elevations in the intracranial pressure. Traumatic brain injury (TBI), neoplasms, subdural, epidural, or intraparenchymal hematomas, acute diffuse cerebral edema, hyponatremia, or administration of thrombolytics may precipitate intracranial hypertension [1-4].

Duret hemorrhages have been invariably associated with devastating prognosis, thereby, dictating withdrawal of care most often [5,6]. Moreover, distinguishing Duret hemorrhages from primary brainstem hemorrhages may be challenging in several instances due to the occurrence of the hemorrhages even within half an hour of the initial trauma [7]. However, recent reports suggest hope for rehabilitation from clinical pessimism caused by an age-old prophecy regarding the outcomes.

Still, the outcome has been so diverse in the previous reports contained in the literature on Duret hemorrhages, that more attention in the reporting of cases is required for a statistical or reasonable assertion of the prognosis of the condition [8-10].

To the best of our knowledge, only four cases of occurrence of Duret hemorrhages post-evacuation of epidural hematoma have been reported. Interpretations on the prognosis of such occurrences and thereby lines of strategic management can only be drawn after a significant number of such cases are reported. We thus add another rare case of occurrence of Duret hemorrhage that occurred in a patient after the evacuation of epidural hematoma.

## Case presentation

**Figure 1:**
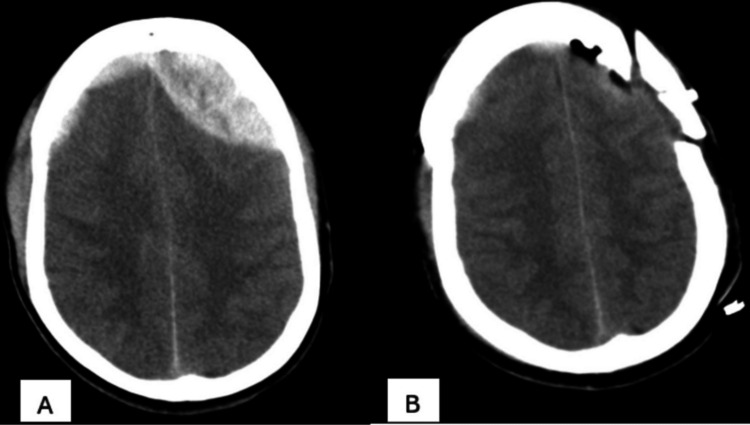
(A) Plain CT scan of brain showing bifrontal acute extradural hematoma, possibly due to shearing pressure on anterior superior sagittal sinus. (B) Craniotomy flap seen with complete evacuation of acute extradural hematoma.

A 20-year-old male presented to the emergency care unit of our hospital with a history of road traffic accidents. On evaluation, he had a Glasgow Coma Score of 10 with pupillary anisocoria. Urgent computed tomography was done, which showed bifrontal extradural hemorrhage with an underlying mass effect, mainly on the left side (Figure 1A). The family was counseled regarding the need for urgent evacuation, to which they agreed immediately. The patient underwent left frontal craniotomy and evacuation uneventfully (Figure 1B).

**Figure 2:**
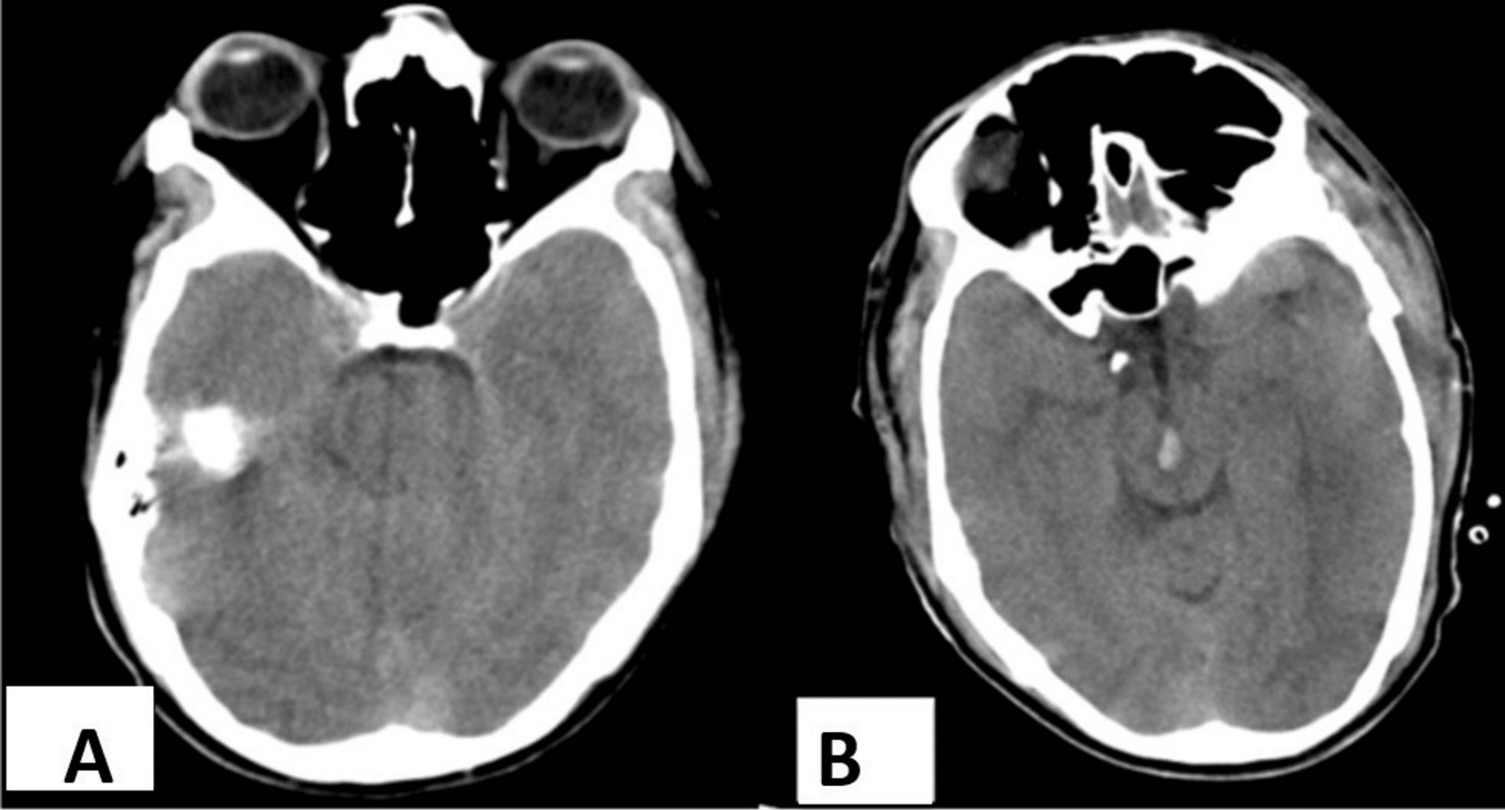
Comparative plain CT scan of brain. (A) Before surgery focusing on brainstem showing no signs of hemorrhage. (B) After surgical evacuation, due to possible descending transtentorial herniation, a newly developed Duret hemorrhage.

Preoperative scans were compared for the same, which showed no signs of brainstem injury (Figure 2A). Postoperatively, his condition remained in a poor neurological state. A repeat scan within 24 hours, was done in view of it, which showed complete evacuation of the hematoma with surprisingly newly developed Duret hemorrhage (Figure 2B).

In accordance with the family’s wishes, aggressive TBI monitoring and treatment in the intensive care unit were continued even though the anticipated outcome was poor. After a lengthy hospital course, the patient remained to be in a vegetative state and was discharged with a Glasgow Outcome Scale score of 2.

## Discussion

Duret hemorrhages were first observed by Henry Duret in his experiments on canid mammals when he injected gelatin or water inside the skulls [11]. Epidural, subdural, or intraparenchymal hematomas lead to a raised intracranial pressure and thereby an inferior push of the brainstem, leading to stretching of the relatively immobile basilar artery and spasm, infarction, and hemorrhage. Duret hemorrhages usually initiated in the midline of the mesencephalon and upper pons result in sideways compression and thereby an anteroposterior elongation of the brainstem and further stretching of the pontine perforating arteries [12,13].

The incidence of them could have possibly been miscalculated due to underreporting of the cases, missed diagnoses on initial emergency CT, posterior fossa artefacts seen on CT, or the microscopic nature of the lesions [13-15]. Duret hemorrhages need to be differentiated from petechial or hypertensive brainstem hemorrhages, traumatic brainstem hemorrhages due to direct, penetrating, or shearing injuries, and ruptured arteriovenous malformations [5].

A systematic review and meta-analysis on Duret hemorrhages after transtentorial brain herniation has mentioned that 63% of the patients in the published case reports have been diagnosed with subdural hematomas [16]. Reports relating to the occurrence of these after evacuation of epidural hematomas are rare, and therefore the traditional belief of mortality related to Duret hemorrhages unfortunately gets universally applied in every case, potentially influencing treatment decisions negatively.

In our case, the patient developed a Duret hemorrhage post-evacuation of epidural hematoma and was discharged in a vegetative state. In the case reported by Fujimoto et al., a 44-year-old female patient who presented with Duret hemorrhage due to transtentorial herniation by epidural hematoma was discharged with minimal neurological defects after two weeks in a coma. The patient survived it despite ocular bobbing, a sign of poor prognosis. The authors recommended rigorous neurological observation and early detection of such hemorrhages to reduce the chances of a bad outcome [17].

Likewise, another case of survival was reported by Stiver et al., wherein a 24-year-old woman suffered a TBI in an accident. The patient had a low Glasgow Coma Scale score and advanced signs of cerebral herniation. She developed hemorrhage post-evacuation of an acute epidural hematoma, which was considered a terminal and irreversible event; however, following her family’s wishes, aggressive neurological monitoring and treatment were continued in the intensive care unit. The patient showed a marked improvement with a Glasgow Coma Scale score of 14 and was discharged ambulatory with good cognitive functioning. The authors opined that continued and aggressive neurological care may change the age-old notion of fatality or persistent vegetative state associated with them, given a careful patient selection or identification who would likely benefit from such prolonged and intensive management and monitoring of prognostic biomarkers, metabolic signatures on neuroimaging, and specific and sensitive neurophysiological tests [18].

Unlike the reports of survival, a report presented by Jost and Taub of a 58-year-old male patient, who developed Duret hemorrhage after the rapid evacuation of epidural hematoma due to TBI exhibited a poor prognosis, with the patient being declared brain dead. The authors suggested that patients who can be instituted with craniotomy and hematoma evacuation in the window period, which is the time between the first-comatose period due to trauma and the second-comatose period due to rising intracranial pressure, may have better chances of survival [19].

Likewise, in a case report by Tyngkan et al., a 25-year-old male patient who suffered from TBI due to a fall from height died five days after he underwent craniotomy and evacuation of extradural hematoma. The patient, although recovered from anisocoria, his Glasgow Coma Scale score did not improve, and the occurrence of duret hemorrhage was evident on a succeeding CT performed postoperatively. The CT scan that was performed within an hour of the injury did not reflect any such brainstem hemorrhage and the patient was operated on within one and a half hours of the injury. However, the authors, in this case, believed this event of hemorrhage was possibly due to a reperfusion injury, as also mentioned in previous reports of such hemorrhages after surgical decompression, rather than tentorial herniation [8]. To the best of our knowledge, reports on the occurrence of Duret hemorrhages linked to epidural hematomas are scarce and we report a fifth case in order.

The outcome of Duret hemorrhages may, although reported to be fatal and an ominous sign in patients undergoing neurosurgery, the condition must not fetch a clinical nihilism and thereby discourage management and care. A diligent reporting of the cases, especially those due to causes that have been rarely associated or reported in the literature, like epidural hematoma or brain neoplasms, might help in drawing more rationale conclusions on the prognosis of patients and thereby more refined dictation of case selection for continuation or institution of aggressive neurological management and care [20].

## Conclusions

Duret hemorrhages have traditionally been considered signs of terminal and irreversible brain damage with a high fatality rate. Literature holds four case reports of Duret hemorrhages after the evacuation of epidural hematomas with variable outcomes, and to attempt to deepen the insight into the conclusiveness of the prognosis of such fatal hemorrhages, we have added a fifth case to the literature. Inclusive of our case, two out of five patients reported with Duret hemorrhages after evacuation of epidural hematoma had a fatal outcome, with the rest of the patients being discharged either in a vegetative state, which was in our case, or with few neurological deficits.
